# AM-Induced Alteration in the Expression of Genes, Encoding Phosphorus Transporters and Enzymes of Carbohydrate Metabolism in *Medicago lupulina*

**DOI:** 10.3390/plants9040486

**Published:** 2020-04-10

**Authors:** Andrey Yurkov, Alexey Kryukov, Anastasia Gorbunova, Andrey Sherbakov, Ksenia Dobryakova, Yulia Mikhaylova, Alexey Afonin, Maria Shishova

**Affiliations:** 1 Laboratory of Ecology of Symbiotic and Associative Rhizobacteria/All-Russia Research Institute for Agricultural Microbiology, 196608 Saint Petersburg, Russia; rainniar@rambler.ru (A.K.); gorbunova.anastasia93@mail.ru (A.G.); microbe-club@inbox.ru (A.S.); 2 Department of Geobotany and Plant Ecology/Saint Petersburg State University, 199034 Saint Petersburg, Russia; 3 Laboratory of Molecular and Environmental Physiology/Komarov Botanical Institute of the Russian Academy of Sciences, 197376 Saint Petersburg, Russia; kdobryakova@mail.ru; 4 Laboratory of Biosystematics and Cytology/Komarov Botanical Institute of the Russian Academy of Sciences, 197376 Saint Petersburg, Russia; ymikhaylova@binran.ru; 5 Laboratory of Genetics of Plant–Microbe Interactions/All-Russia Research Institute for Agricultural Microbiology, 196608 Saint Petersburg, Russia; afoninalexeym@gmail.com; 6 Department of Plant Physiology and Biochemistry/Saint Petersburg State University, 199034 Saint Petersburg, Russia; mshishova@mail.ru

**Keywords:** arbuscular mycorrhiza, sugar metabolism, phosphorus transporter, gene expression, *Medicago lupulina*, *Rhizophagus irregularis*, efficient symbiosis

## Abstract

Plant–microbe interactions, including those of arbuscular mycorrhiza (AM), have been investigated for a wide spectrum of model plants. The present study focuses on an analysis of gene expression that encodes phosphate and sugar transporters and carbohydrate metabolic enzymes in a new model plant, the highly mycotrophic *Medicago lupulina* MLS-1 line under conditions of phosphorus deficiency and inoculation with *Rhizophagus irregularis*. Expression profiles were detected by RT-PCR at six plant stages of development (second leaf, third leaf, shooting, axillary shoot branching initiation, axillary shoot branching, flowering initiation). In comparison to control (without AM), the variant with AM inoculation exhibited a significant elevation of transcription levels of carbohydrate metabolic enzymes (MlSUS, MlHXK1) and sucrose transporters (MlSUC4) in *M. lupulina* leaves at the shooting stage. We suggest that this leads to a significant increase in the frequency of AM infection, an abundance of mycelium in roots and an increase in AM efficiency (which is calculated by the fresh weight of aerial parts and roots at the axillary shoot branching initiation stage). In roots, the specificity of *MlPT4* and *MlATP1* gene expressions were revealed for effective AM symbiosis. The level of *MlPT4* transcripts in AM roots increased more than tenfold in comparison to that of non-specific *MlPT1* and *MlPT2*. For the first time, *MlPT1* expression was shown to increase sharply against *MlPT2* in *M. lupulina* roots without AM at the shooting initiation stage. A significant increase in *MlRUB* expression was revealed at late stages in the host plant’s development, during axillary shoot branching and flowering initiation. The opposite changes characterized *MlHXK1* expression. Alteration in *MlHXK1* gene transcription was the same, but was more pronounced in roots. The obtained results indicate the importance of genes that encode phosphate transporters and the enzymes of carbohydrate metabolism for effective AM development at the shooting stage in the host plant.

## 1. Introduction

More than 80% of plant species can form arbuscular mycorrhiza (AM) with the Glomeromycotina subdivision of the Mucoromycota division in native ecosystems [1]. Nowadays, AM is being actively introduced in agricultural systems. Thus, the investigation of the mechanisms responsible for the symbiotic efficiency of plant interaction with AM fungi has both theoretical and practical importance. Molecular, physiological and biochemical approaches are widely employed with the aim of revealing these mechanisms.

In AM symbiosis, the host plant supplies AM fungus with photosynthates in the form of hexose [2] and, to a lesser extent, sucrose [2,3,4]. The plant, in turn, receives water and a number of macro- and microelements, especially phosphorus [5]. Thus, AM efficiency depends on the intensity of phosphate and carbohydrate transport processes between the partners. Alteration in the carbohydrate/mineral nutrient exchange might convert the plant–microbial interaction from a mutualistic to a parasitic one, which might affect the intensity of host plant development [5,6,7].

Plant growth depression was shown for spring wheat plants (*Triticum aestivum*) inoculated with *Glomus intraradices* (DAOM181602 strain, now classified as *Rhizophagus irregularis*) and *Gigaspora margarita* (WFVAM21 strain) in comparison to control plants without AM under conditions of different levels of phosphorus deficiency in the substrate. The elevation of AM efficiency (reduction in the growth depression) was detected with a rise in plant density per pot. It was supposed that such increasing AM efficiency was determined not only by less competition for soil phosphorus in AM plants (and greater competition in plants without AM), but also by reducing the cost of carbohydrate transfer to AM fungi interacting with a greater number of plants [7]. Thus, the intensity of supply in between the host plant and the microorganism contributes to the regulation of AM symbiotic efficiency. The importance of phosphate and carbohydrate transporters as well as enzymes of carbohydrate assimilation are of great interest for the investigation of efficient AM symbiosis formation and development. Regulation of the proteins’ activity appeared at different levels, including at the transcriptional level. The identification of genes encoding enzymes of carbohydrate metabolism, sugars and phosphate transporters, as well as the investigation of its expression, are major priorities for contemporary AM research.

### 1.1. Phosphate Transport in AM Plants

Soil phosphates are insoluble which makes them a limiting factor for plant growth. AM symbiosis is beneficial for plants because AM fungi absorb inorganic phosphorus from the soil, help transform it into polyphosphate, accumulate it, move it through hyphae, hydrolyze it, and then transport it to plant cells in the form of phosphate anions. Inside the root, AM fungi are represented as the mycelium, the vesicles, and the arbuscules. The latter is believed to play a key role in the mutual nutrition and signal exchange between partners [5]. It was shown that the formation of AM increases the intensity of phosphorus uptake by the roots of the host plant by two to four times, whereas 80% of phosphorus is absorbed by mycosymbionts [8]. AM fungi efficiently assimilate phosphates because of their high adsorption of mycelium and the fact that active excretion of organic acids and, in particular, phosphatases result in its ability to dissolve unavailable phosphorus [9]. The elevation of phosphorus uptake into AM plants is related to the activity of AM-specific phosphate transporters of the PHT1 family [5,10]. PHT1 transporters belong to the phosphate transporters of the H^+^-symporter family, PHS [11]. For example, MtPT4 is a key phosphate transporter from AM fungi to *M. truncatula* [12]. *MtPT4* gene expression was discovered only in cells with arbuscules. The level of expression increased during AM development [13]. On the contrary, the expression of *MtPT1* was suppressed in the presence of AM or at high phosphorous levels. The MtPT1 protein is localized in root epidermal cells. It is the main plant transporter of phosphorus uptake directly from the soil. A decrease in the amount of MtPT1 protein in the roots of *M. truncatula* with AM indicates that this transporter is not involved in symbiotic phosphate transport [14]. *MtPT2* gene expression is also significantly suppressed with high levels of phosphorus in the substrate. The accumulation of *MtPT3* gene transcripts decreases both in the presence of phosphorus and AM fungi. However, AM scarcely reduces the expression of the *MtPT5* gene [12]. Despite the recent presentation of the data dynamics of *MtPT6-MtPT9* [15,16,17] and *MtPT10-MtPT13* [18], the nature of their expression is still puzzling.

A number of proteins were also shown to modulate the intensity of phosphate transport. The H^+^-ATPase (ATPase1, MtHA1) was found to be specific for AM and important due to the necessity to generate a proton gradient required for phosphate transport through the periarbuscular membrane [9,19,20]. PHR1 [21,22,23] and CBX1 [24] transcription factors regulate the activity of genes involved in the efficient transport of phosphate in AM structures. CBX1 presumably regulates the genes encoding AM-specific transporters of the PHT1 family (e.g., MtPT4) and H^+^-ATPase (e.g., MtHA1), as well as proteins involved in fatty acid biosynthesis [24].

Many of the mechanisms of phosphate transport in AM are still poorly understood. Data is still inadequate on the expression of the transporter genes of the PHT1 family and the mechanisms regulating their expression.

### 1.2. Carbohydrate Metabolism of AM Plants

Plant carbohydrate metabolism was shown to change significantly during AM formation. The host plant transfers up to 30% of assimilates towards the AM fungus [2,25]. Besides carbohydrates, it donates other metabolites, such as fatty acids, known to be used as a source of organic carbon directed in the synthesis of fungal lipids [26]. AM affects photosynthesis activity (increase in chlorophyll content, leaf surface area, and CO_2_ fixation rate) and enhances the photoassimilates’ flux towards host roots [5]. A similar complex effect is never obtained with the phosphorus fertilizer treatment. It is presumed that plants receive better benefit by effective symbiosis with AM fungi than by developing an equivalent level of root absorption.

The assumption is that AM affects the activity of symbiosis specific plant genes encoding metabolic enzymes and sugar transporters [27,28,29]. For example, in the case of symbiotic sucrose synthase *MtSucS1* knockdown, the development of arbuscules in *M. truncatula* was inhibited [30]. However, little is known about the dynamics of the expression of genes encoding such important enzymes as ribulose-1,5-bisphosphate carboxylase/oxygenase (RUBISCO) and hexokinases in mycorrhizal plants. The hexokinase family of enzymes is involved in glucose phosphorylation; RUBISCO is responsible for carbon dioxide fixation in the Calvin cycle [31,32]. Such a lack of detailed information might be due to the complications of discovering specific pathways which integrate transport, deposition and catabolism during AM formation.

Plant sugar transporters are integrated in three key families: the sucrose/H^+^-symporter Sucrose Transporter (SUT), Monosaccharide Transporters (MST), including the Sugar Transport Protein subfamily (STP), etc., and Sugars Will Eventually be Exported Transporters (SWEETs). For some plant species, Sucrose Facilitator (SUF) and Chloroplast Maltose Exporter (MEX) transporters have additionally been identified [33]. These transporters have been shown to be involved in the transport of sugars in AM plants. An increase in Mtst1 (MtMST family) accumulation during mycorrhization has been previously shown [34]. Subsequent investigation has shed some light on the common mechanisms of sugar transport. These include: detection and analysis of sink cell hexose transporters MtMSTs in *Medicago truncatula* [27,35], the sucrose transporters SlSUTs in *Solanum lycopersicum* during mycorrhization (e.g., low expression of *SlSUT2* leads to higher mycorrhization) [29,36], *StSUT1* in *S. tuberosum* (overexpression of the gene leads to increased mycorrhization) [29,37], as well as *SUT4* [29] and genes of the SWEET family, *StSWEET2c*, *StSWEET7a*, *StSWEET12a* in *S. tuberosum* (which have specific localization in arbuscular-containing root cells) [29,38] and *LjSWEET3* in *Lotus japonicus* (increased expression in AM) [29,39]. Expression of the genes from the MtSTP family was revealed in arbuscule-containing cells of *M. truncatula* [29]. Thus, AM induces alterations in the representation of sugar transporters from different gene families; however, they are not well documented [40].

There are still gaps in the scheme of carbohydrate metabolism and phosphate/carbohydrate transport in plants with and without AM fungi [33]. New approaches such as transcriptomic, proteomic and metabolic analyses are used in recent studies. Thus, new data focusing on key enzymes and transporters in plants forming AM are anticipated.

The aim of this study is to evaluate the expression of genes encoding sugar and phosphate transporters, as well as important carbohydrate metabolism enzymes. The expression profile was detected in the plants of the *Medicago lupulina* MlS-1 line with and without *Rhizophagus irregularis* inoculation of AM fungi that were highly responsive to mycorrhization (highly mycotrophic). The comparison was carried outduring host plant development under conditions of low phosphorous content in the substrate.

## 2. Results

**Figure 1:**
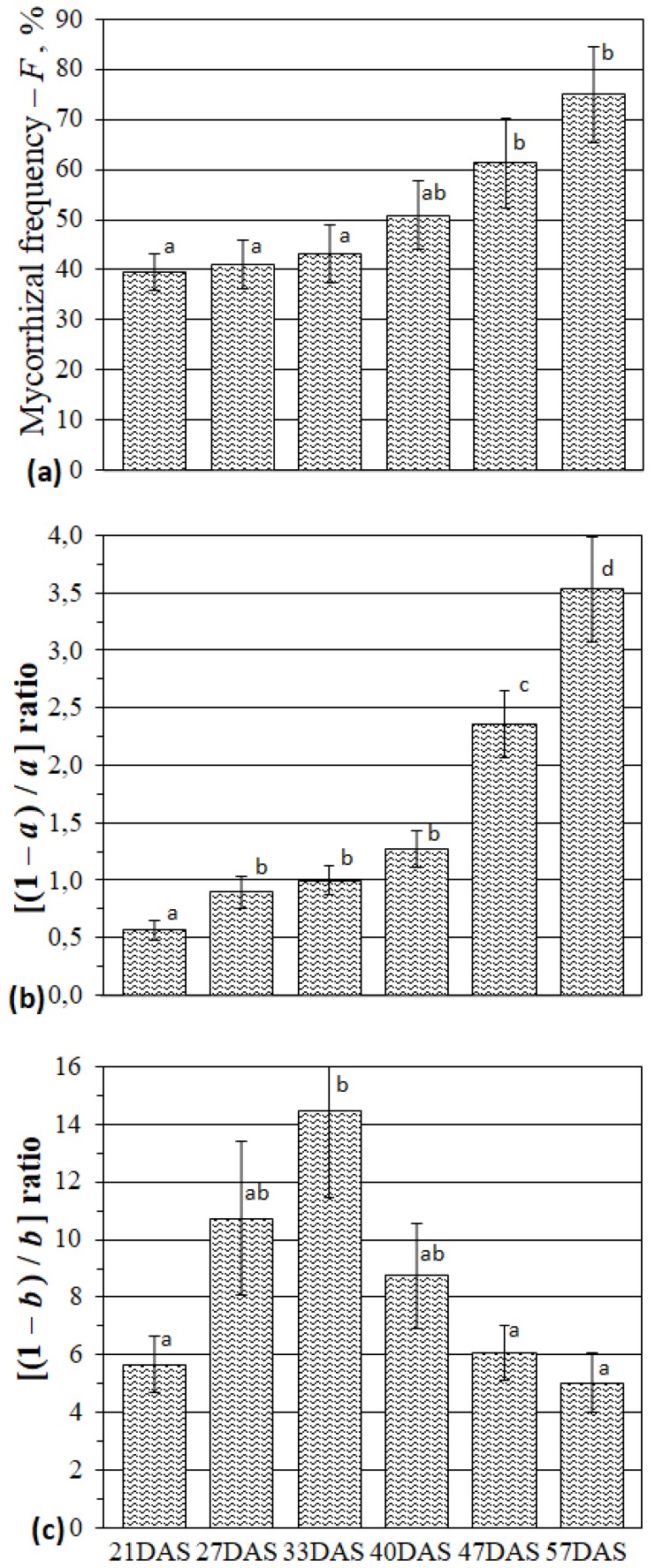
The mycorrhization parameters for *M. lupulina* roots: mycorrhizal frequency (**a**); ratio of mycelium abundance without arbuscules to arbuscule abundance, [(1 − *a*)/*a*] (**b**); ratio of mycelium abundance without vesicules to vesicule abundance, [(1 − *b*)/*b*] (**c**). Day after sowing (DAS). Different letters indicate significant differences within the same mycorrhizal parameter (ANOVA and Tukey’s test; *p* < 0.05). The average values (means of eight replicates) with standard errors are presented.

The analysis of mycorrhization parameters of the *M. lupulina* MLS-1 plant line with a highly intensive response to AM inoculation showed that the frequency of mycorrhizal infection (*F*) even at the second true leaf stage of plant development (2L) (for abbreviations for stages of development see Table 1), at 21 days after sowing (DAS), was already 39.6% and increased by 1.9 times up to FI (57 DAS) (Figure 1a). The abundance of mycelium without arbuscules is expressed by the “[100% − *a*]” value, another important parameter “[1 − *a*]/*a*” reflects the ratio of the mycelium abundance without arbuscules to the abundance of arbuscules in the root. The “[(1 − *a*)/*a*]” value (Figure 1b) rose substantially during development: slowly (1.8 times) up to 33 DAS (shooting (SH) stage of *M. lupulina* plants) and more sharply (3.5 times) up to 57 DAS (at FI). We observed an increase in the abundance of mycelium without vesicles in comparison with the abundance of vesicles. The parameter “[(1 − *b*)/*b*]” reflects the ratio of the mycelium abundance without vesicles to the abundance of fungal vesicles in the root. Its value rises by 2.6 times up to SH and is followed by a significant (*p* < 0.05) decrease from 33 DAS to 57 DAS at FI (Figure 1c). The latter stage of plant development is characterized by an elevation in the abundance of mycelium without vesicles which was five times higher than that with vesicles.

**Figure 2:**
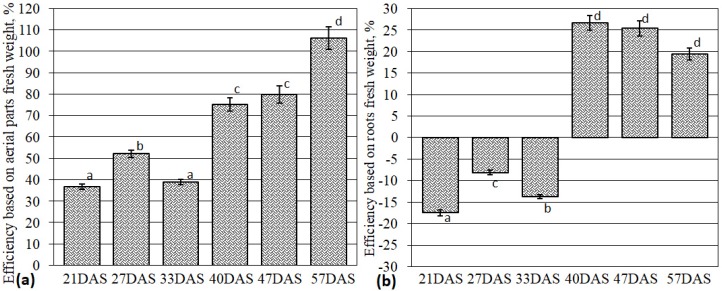
Arbuscular mycorrhiza (AM) symbiotic efficiency calculated by *M. lupulina* shoots (aerial parts) fresh weight (**a**) and roots fresh weight (**b**). Different letters indicate significant differences within the same efficiency parameter (ANOVA and Tukey’s test; *p* < 0.05). The average values (means of eight replicates) with standard errors are presented.

AM efficiency was calculated as a ratio of the difference in fresh weight between mycorrhized plants (+AM) and control plants (−AM) to that of the control variant. Significant increases in AM efficiency were observed (1.9 times from 33 DAS to 40 DAS; Figure 2a). Further on, an increase from 40 DAS to 57 DAS was determined (1.4 times; Figure 2a). At the same time, dramatic changes were detected in the AM efficiency calculated as function of the fresh root weight (Figure 2b). Control plants (−AM) had a more developed root system at earlier stages (including 33 DAS). Active root growth was observed in plants with AM starting from SH. Significant AM efficiency was shown at the next stage of *M. lupulina* development (axillary shoot branching initiation (SBI) at 40 DAS). AM efficiency for fresh root weight was maintained until the last stage of analysis, flowering initiation (FI).

The alteration in AM efficiency is linked to the intensity of phosphate and carbohydrate exchanges between the host plant and AM fungus. This investigation is focused on the expression of genes encoding representatives of the transporters involved in the exchange. The genes were selected and primers for these genes were designed (Table 2). Sequence data was obtained from the early performed transcriptome assembly of the analyzed highly responsive *M. lupulina* MLS-1 line within the frame of a separate investigation (SUBID: SUB6631257; BioProject: PRJNA592725; BioSample: SAMN13439142; Accession: GIDJ00000000; Organism: *Medicago lupulina*).

**Figure 3:**
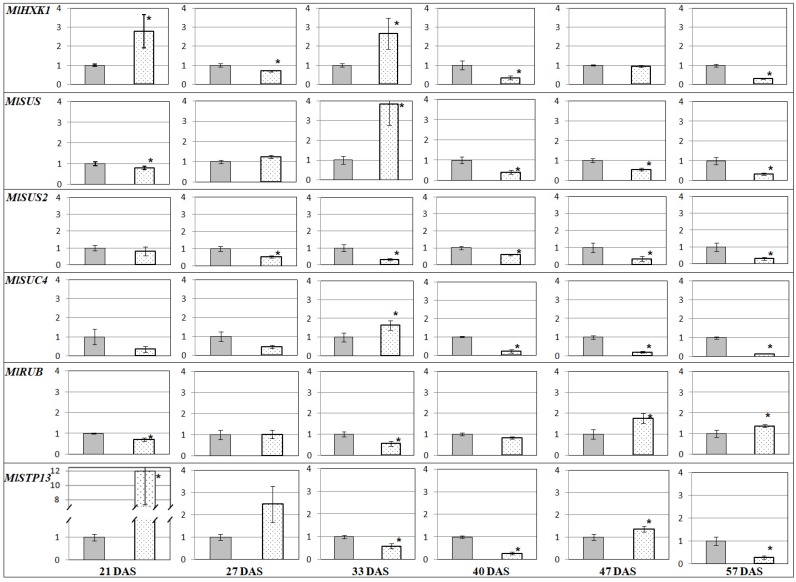
Relative transcript levels (2^−ΔΔCt^ normalized values) in *M. lupulina* leaves. The average values (means of three replicates) with standard errors in the experiment with AM plants against control plants without AM (gray bar) are presented. * —significant (*p* < 0.05) differences using the *t*-student test. *MlPT1*, *MlPT2*, *MlPT4* and *MlATP1* genes not expressed.

**Figure 4:**
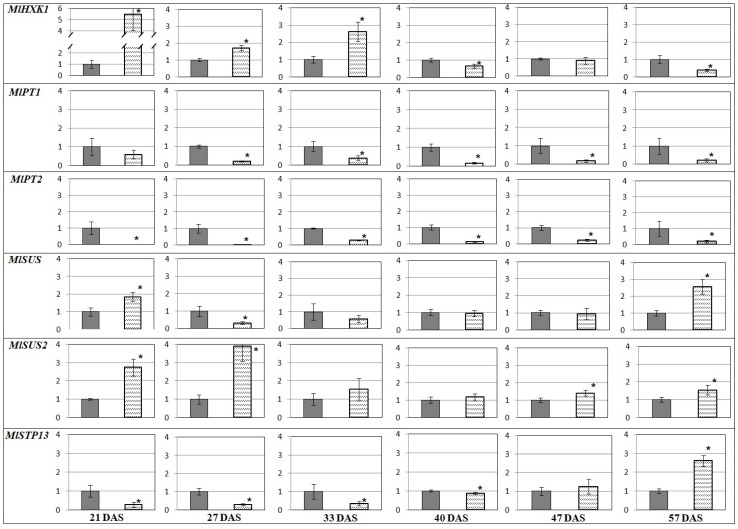
Relative transcript levels (2^−ΔΔCt^ normalized values) in *M. lupulina* roots. The average values (means of three replicates) with standard errors in the experiment with AM plants against control plants without AM (gray bar) are presented. * —significant (*p* < 0.05) differences using the *t*-student test. *MlPT4* and *MlATP1* genes were specifically expressed in roots with AM and therefore were not presented. *MlSUC4* expression was very low in roots with/without AM and therefore was not analyzed.

A comparison of the transcription profiles revealed that genes encoding the sugar transporter (*MlSUC4*) and the enzymes of carbohydrate metabolism (such as *MlSUS* and *MlHXK1*) had higher expression in AM plant leaves vs. the control (−AM) at SH (33 DAS; Figure 3). In roots, transcript levels were higher at the beginning (*MlHXK1*, *MlSUS, MlSUS2*) and the end of vegetation (*MlSUS, MlSUS2*, *MlSTP13*). *MlPT1* and *MlPT2* genes were continuously expressed at higher levels in plants without AM (Figure 4). The obtained results showed that the expression of genes encoding the RUBISCO small subunit (*MlRUB*) had complex dynamics (Figure 3). In our experiments, the accumulation of *MlRUB* transcripts was less intense in AM plant leaves at the initial stages of *M. lupulina* development (up to SH, 33 DAS). Then, up to SBI at 40 DAS, the *MlRUB* transcript level in AM plant leaves was aligned with the transcript level in the control plants, becoming higher at SB (47 DAS) and FI (57 DAS). In the latter stages of development, carbon assimilation apparently prevails over its degradation, because the decrease in *MlHXK1* and increase in *MlRUB* transcript levels against control (−AM) in the latter stages of development (40–57 DAS; Figure 3).

The highest *MlHXK1* transcript accumulation both in leaves (Figure 3) and in roots of AM plants (Figure 4) was estimated at 21 DAS and, at SH, 33 DAS.

**Figure 5:**
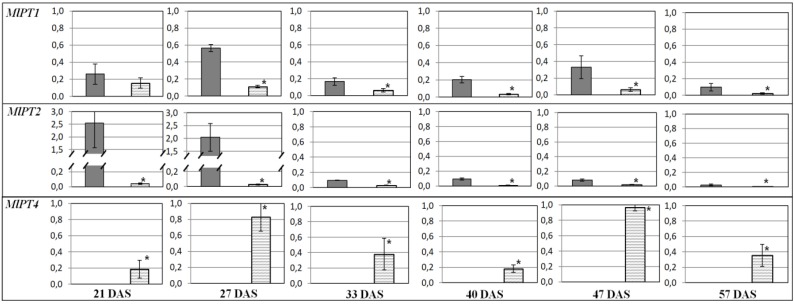
Relative transcript levels (2^−ΔCt^ normalized values) of genes from the same PHT1 gene family in *M. lupulina* roots. * —significant (*p* < 0.05) differences using the *t*-student test. *MlPT4* was specifically expressed in plants with AM.

Expressions of genes encoding sucrose synthases, *MlSUS* and *MlSUS2*, in leaves had similar dynamics, except SH (33 DAS). The number of *MlSUS* transcripts was significantly larger than *MlSUS2* transcripts. From SH, the expressions of both genes were significantly (*p* < 0.05) lower in the leaves of AM plants compared to the control plants (‒AM) (Figure 3). However, in roots, the expressions of these genes, on the contrary, were aligned at SBI and after. At FI, the sucrose synthase transcription levels in leaves of AM plants exceeded those in the control (Figure 4). The sucrose-H^+^ symporter gene *MlSUC4* was less transcripted in leaves of AM plants throughout development, except SH at 33 DAS (Figure 3). Accumulation of *MlSTP13* transcripts, the gene encoding the monosaccharide-H^+^ symporter, was also low and characterized by complicated dynamics in both the leaves and roots (Figure 3 and Figure 4). The first significant decrease in *MlSTP13* expression in leaves of AM plants against control was observed at SH. In contrast, *MlSTP13* expression levels in AM plant roots were less intensive than in the control at the initial stages of development (21–33 DAS), but expression levels then increased up to SBI (40 DAS). It was shown that the expression of *MlPT1* and *MlPT2* genes (*P_i_*-transporters genes) was significantly weaker in the roots of AM plants at all stages of *M. lupulina* development (Figure 4). Presumably, *P_i_* uptake in conditions of low *P_i_* content in the substrate was mainly due to transporters specific of AM. *MlATP1* (gene encoding H^+^-ATPase) and *MlPT4* (*P_i_*-transporter gene) are presumably involved in *P_i_* transport across the periarbuscular membrane in cells of *M. lupulina* roots interacting with *R. irregularis* AM fungi. Since *MlPT1*, *MlPT2* and *MlPT4* belong to the same PHT1 gene family (like homologs in *M. truncatula*), we assumed that it is possible to compare the levels of their transcripts, normalized to the common reference gene (Figure 5). Such an approach of data comparison permitted highlighting the contribution of *MlPT2* transcripts in *M. lupulina* roots without AM (between four and 10 times more than *MlPT1* transcript levels at 2L and 3L stages). It changed to a higher contribution of *MlPT1* transcripts (two to four times more than *MlPT2* transcript levels) with a key shift at SH (33 DAS) and with a maximum at SBI. On the other hand, in inoculated plants at the 2L stage (21 DAS), the symbiotrophic *P_i_* uptake was fulfilled by the MlPT4 transporter. Its transcription was probably at the level of the non-symbiotrophic *P_i_* uptake with the participation of MlPT1 and MlPT2 transporters. However, the proportion of *MlPT4* transcripts increased dramatically from 48% to 85% at the 3L stage, and grew until FI (57 DAS), when it reached 93% (almost 15 times more than total *MlPT1* and *MlPT2* transcript levels; see Figure 5). Thus, it can be supposed that the main *P_i_* transport in *M. lupulina* roots occurred in a symbiotic way from *R. irregularis* AM fungi directly through the detected MlPT4 transporter.

## 3. Discussion

An analysis of the AM symbiotic efficiency in *M. lupulina* with *R. irregularis* showed significant (*p* < 0.05) decreases in the SH stage (Figure 2). Such a decrement might be caused by the necessity of energy and metabolites supporting developmental processes for the host plant. According to our earlier data, *M. lupulina’s* SH stage was characterized by a maximum increase in symbiotrophic *P_i_* uptake, both in leaves and roots [41]. Further plant development from SH to SBI was accompanied by the sharp increase in symbiotic efficiency in the weight of both aerial parts and roots. Thus, *M. lupulina* plants achieved the most effective interaction with *R. irregularis*. The root weight efficiency reached the maximum (Figure 2b), while the weight of the aerial parts continued to grow (Figure 2a). Therefore, it can be expected that SH stage can be characterized by the key changes in the AM plants’ metabolism. Note that *F* did not change much before SH (33 DAS) (Figure 1a), but then elevated significantly. *Zea mays* was characterized by similar *F* alterations during stem development [42]. *F* is a complex parameter of AM development, which indicates that host plants from SH begin to accelerate development of AM fungi’s symbiotic structures, especially mycelium.

The abundance of intraradical mycelium “[1−*a*]” exceeds the abundance of arbuscules (*a*) in the latter stages of AM development after the SH stage (40–57; Figure 1b). Mycelium in roots can directly participate in *P_i_* transfer to plants (the *P_i_* level in intercellular hyphae was significantly higher than the *P_i_* level in arbuscules) [43] and sucrose flux to AM fungi [44]. The possibility of active metabolic processes in symbiotic mycelium might be realized by diverse types of AM—for instance, the *Paris* type [45]. A significant role of mycelium in the effective AM symbiosis in the latter stages was shown for symbiosis of *M. lupulina* with *R. irregularis* [41]. The expression of sucrose fungal transporter gene *MST2* (in *M. truncatula* + *Glomus sp.* symbiotic system) was determined not only in arbuscules but also in the intercellular hyphae, which indicated that both structures appear to be important for sugar unloading [44]. The mycelium of the AM fungi probably receives carbohydrates from the apoplast of the root cortex [5] as glucose [46], and less as sucrose [2]. The exact location and mechanism of carbon transfer from host plants to AM fungi is not yet fully discovered, but it is the focus of recent studies of plant–microbial symbiotic interactions [17,18,27,28,29,33].

Carbohydrate metabolism starts with sugar biosynthesis, so it is important to analyze the possible alteration in the expression of genes encoding RUBISCO subunits. This study focused on the expression of the *MlRUB* gene, encoding the small RUBISCO subunit (*rbcS* = *RUB*). *rbcS* plays a role in the regulation of the enzyme activity [47]. *MlRUB* was intensively expressed in both types of *M. lupulina* leaves (+/−AM). A slight decrease in its expression was detected during SH in AM plants vs. the control. At this stage (33 DAS), the control plants intensively developed (Figure 2) and, presumably, some energy resources were directed towards plant organ formation. This stage was also characterized by the intensive formation of effective AM. Later on (at SB and FI stages), the transcript level for *MlRUB* increased significantly (Figure 3), ensuring the maintenance of highly effective AM symbiosis. This might reflect the fact that the presence of AM intensified accumulation of chlorophyll and photosynthesis activity in *M. lupulina* [48].

HXK1 is a vital enzyme localized in cytosol on the outer membrane of the mitochondria in eudicots and in chloroplasts in source cells, as well as in cytosol and apoplasts in sink cells [49]. It should be noted that hexokinase, in addition to the usual function of redistribution of the glucose, also performs a signaling function, mediating the glucose-induced repression of genes associated with photosynthesis, such as *rbcS* [49,50,51]. The changes in *MlHXK1* transcript accumulation are opposite to the dynamics of *MlRUB* transcript levels in leaves during host plant development (a distinct inverse relationship was revealed), a fact which is in agreement with some earlier results [51]. Unfortunately, there are no published data on the dynamics of *rbcS* and *HXK1* expression levels in the other leguminous plants. However, it was shown that overexpression of the *HXK1* gene in *Arabidopsis*, tomatoes and rice results in lower growth intensity, lower chlorophyll content, suppression of photosynthesis and negative impacts on *rbcS* expression [51].

A key result was that the *MlHXK1* had similar alterations in the leaves and roots of AM plants (Figure 3 and Figure 4), which emphasizes the importance of the enzyme both for roots and aerial organs. At the SH stage, the number of *MlHXK1* transcripts was significantly higher (almost 2.8 times) in AM plants compared to the control (Figure 3 and Figure 4). Higher intense sucrose transfer toward *R. irregularis* might be due to the active development of mycelium ([1−*a*]; Figure 1b) and other symbiotic structures at the SH stage (*F*; Figure 1a). The reduction in *MlHXK1* transcript levels in *M. lupulina* AM roots vs. the control (−AM) (Figure 4) after the SH stage may be associated with a decrease in glucose unloading to amyloplasts. The amyloplasts change proliferation in the presence of arbuscules [52,53]; the number of amyloplasts in cells with arbuscules was either greater (*Z. mays*, *Vitis vinifera* [52,54,55]), or less (*Lotus japonicus* [56]) depending on the presence of the former. Thus, the number and distribution of amyloplasts in the root cortex cells with arbuscules are species-specific parameters, and it can be assumed that the content of sugars in the cytosol of root cortex cells depends intensively on their metabolism.

An essential enzyme for effective AM development is Sucrose Synthase (SUS), glycosyl transferase, which is believed to play an important role in the formation of uridine diphosphate glucose during cellulose synthesis. The *MlSUS* transcription level increased sharply during plants’ shooting stage with AM vs. the control (Figure 3). In our experiment, this period coincided with significant changes in AM development: (1) *F* significantly increased (Figure 1a); (2) the abundance of mycelium without arbuscules ([1−*a*]) aligned with the abundance of arbuscules (*a*) and continued to grow (Figure 1b). For *MtSucS1* and *MtSucS2*, which are homologs of *MlSUS* and *MlSUS2* in *M. truncatula* [30,57], gene expression was determined only at 50 DAS [30]. The authors suggested: (1) the important role of *MtSucS1* in the maintenance of arbuscules must be taken into account; (2) the *M. truncatula* mutant lines with reduced expression of *MtSucS1* showed abnormalities in their expression of symbiotic *P_i_*-transporter *MtPT4*, and usually formed short-lived arbuscules; (3) the expression of *MtSucS2* was two to three times higher than that of the wild-type line [30]. On the contrary, the highly mycotrophic MLS-1 line of *M. lupulina* in our experiment showed a high AM efficiency and a high level of *MlSUS* transcripts in leaves at SH. Synthesis of SUS is well known to be increased under stress conditions [58]. Therefore, its lower content in the latter stages of *M. lupulina* development might indicate that AM plants completely overcome the *P_i_* deficiency. *MlSUS2* transcript levels in leaves of AM plants are lower at all stages of development (Figure 3), so MlSUS2 is assumed to play a minor role in carbohydrate metabolism in the leaves of *M. lupulina* AM plants.

Nevertheless, *MlSUS2* is expressed more actively than *MlSUS* in AM roots in comparison with the control (‒AM). This may highlight a significant redistribution of sucrose in sink cells and root cells with AM, because SUS catalyze a reversible reaction of synthesis/degradation of sucrose in sugar-consuming root cells [27,59]. According to the literature, genes of the family of sucrose synthases tend to be characterized by increased expression in roots with AM [30]. SUS1 seems to be involved in the induction of arbuscule formation and the maintenance of normal arbuscule maturation and activity [30].

Another method for sucrose reloading in mesophyll cells is possible extrusion from the plant vacuole into cytoplasm due to the sucrose symporter SUC4. The *MlSUC4* showed a large transcript level only during the SH stage, when the intensive development of symbiotic structures was observed (Figure 3). Further higher expression of *MlSUC4* in control plants was associated with phosphate starvation caused by low *P_i_* level in the substrate, and therefore caused conditions where there was a constant lack of organic compounds for the normal development of the host plant. In *M. truncatula*, *MtSUT4-1* (homolog to *MlSUC4*) is of greatest interest. It encodes the sucrose symporter, which is actively accumulated both in root cells with arbuscules and in the cells of leaves. MtSUT4-1 localizes in the tonoplast and is involved in sucrose transfer from vacuole to cytosol [60]. Thus, the sucrose in *M. lupulina* roots can be exported from the vacuole into the cytoplasm by MlSUC4. *MlSUC4* in *M. lupulina* roots is very low. The mechanisms for sucrose unloading in roots (including *SUC4* expression) have been studied very fragmentarily and partially based on indirect modeling [61].

A reverse influx of sugars from the cell wall to cytosol of mesophyll cells can also be provided by STP13. A clear trend in the decreasing transcript level of *MlSTP13* (hexose transporter) was detected in the leaves of *M. lupulina* AM plants vs. the control (Figure 3). In contrast, the transcript level of *MlSTP13* increased significantly in the roots of *M. lupulina*-mycorrhized plants in the latter stages (Figure 4). Such results obtained for *M. lupulina* may indicate an intensive flux of hexoses at the FI stage from the arbuscules of *R. irregularis* to the cytosol of plant cells, or hexose transport from the cell wall space to the arbuscule-containing cells [29].

*P_i_* transfer associated with carbohydrate metabolism is localized mainly in peri-arbuscular space [62]. Specific *P_i_* transporters were found in *M. truncatula*, *Solanum tuberosum*, *Oryza sativa*, and other plants [63,64,65]. This study showed, for the first time, that *MlPT4* expression in *M. lupulina* roots was intensive and AM-specific. Transcript levels of AM-specific *MlPT4* were higher than the levels of both *MlPT1* and *MlPT2* transcripts in *M. lupulina* roots at FI (Figure 5). Our conclusions are that: (1) a significant increase in AM efficiency in *M. lupulina* during SH, SBI and FI (Figure 2) is linked with high *MlPT4* expression in the roots of AM plants. This may be associated with active development of intraradical mycelium (Figure 1b). (2) Mycelium can play an important role in the transport of phosphate and carbohydrates under conditions of low *P_i_* levels in the substrate.

Symbiotic *P_i_* uptake across the periarbuscular membrane requires the generation of a proton gradient by H^+^-ATPase [63,66], namely ATP1 (homolog to MtHA1; in *M. truncatula*, a single isoform, *MtHA1*, was specifically expressed in cortical cells containing arbuscules) [19,20,66]. *MlATP1* expression in *M. lupulina* roots was AM-specific. Upregulation of H^+^-ATPase genes has been reported in the AM roots of several plant species: *M. truncatula*, *Solanum lycopersicum* and *Nicotiana tabacum* [19,67,68].

Nonspecific PT1 and PT2 transporters are not localized in the peri-arbuscular membrane [12,14,66,69]. The transcript levels of *MlPT1* and *MlPT2* in *M. lupulina* AM roots were lower (vs. control) at all stages of development (Figure 5). Starting from the SH stage, these low transcript levels of *MlPT1* and *MlPT2* in AM roots were compensated (in abundance) by the high expression of *MlPT4*. Equally important are the obtained results demonstrating an intensive decrease in the level of *MlPT2* transcripts vs. *MlPT1* in the roots of *M. lupulina* plants without AM from the 2L to 3L stages. The level of *MlPT1* transcripts (vs. *MlPT2*) increased at this stage and continued rising until the SBI stage. The results are consistent with the literature data [12]. Thus, *MtPT1* expression was higher in plants exposed to *P_i_* starvation [14]. At the high levels of *P_i_* in substrate, the expression of *MtPT2* and *MtPT1* genes was significantly inhibited [12,14]. The present study showed that *M. lupulina* plants without AM perhaps experienced an intensive lack of *P_i_*, starting with the 3L stage, and the adaptation to low *P_i_* levels did not occur. *M. lupulina* plants inoculated with *R. irregularis* did not experience a lack of *P_i_* up to the FI stage, as confirmed by the low level of *MlPT1* vs. *MlPT4* transcripts (Figure 5).

## 4. Conclusions

In conclusion, this study was the first to analyze the expression of phosphate transport and carbohydrate transport, as well as carbohydrate metabolism in genes in *M. lupulina* AM plants that formed highly effective symbiosis with *R. irregularis* under conditions of P*_i_* deficiency. Two genes specific for expression in AM, *MlATP1* and *MlPT4*, were identified. The AM plants of *M. lupulina* did not experience *P_i_* starvation, but *M. lupulina* plants without AM did not adapt to the low *P_i_* level in the substrate. For the first time, it was shown that the proportion of *MlPT1* vs. *MlPT2* transcripts in plants without AM was lower at earlier stages of development, but from the stage of shooting initiation (when *P_i_* deficiency begins to manifest itself), the proportion of *MlPT1* vs. *MlPT2* transcripts significantly increased. *MlPT1* can play a key role in the transport of phosphate in *M. lupulina* without AM under conditions of *P_i_* deficit in the substrate. Nevertheless, the transcript levels of genes encoding carbohydrate metabolic enzymes (MlSUS, MlHXK1) and sucrose transporters (MlSUC4) were higher in leaves of AM plants vs. the control plants at the shooting stage (33rd DAS). This was probably associated with a subsequent significant increase in AM efficiency, calculated by the fresh weight of *M. lupulina* plants at the axillary shoot branching initiation stage (40th DAS). Thus, the shooting stage was concluded to be the key point in triggering an efficient interaction between *M. lupulina* and *R. irregularis*.

## 5. Materials and Methods

### 5.1. Biomaterials

In this study, we used the black medic (*Medicago lupulina* L. *subsp. vulgaris* Koch; synonyms: black medick, black medic clover, hop clover, hop medic, nonesuch clover, yellow trefoil) MlS-1 line from VIK32 cultivar-population (cultivar-population selected by G.V. Stepanova from Federal Williams Research Center of Forage Production and Agroecology; MlS-1 line selected by A.P. Yurkov and L.M. Jacobi from ARRIAM) [70]. Black medic is a widespread species of Medicago genus, a self-pollinating diploid (2n = 16) with ~800 Mb genome size and high seed production up to 2500 seeds from one plant. MlS-1 plants have signs of dwarfism in the absence of AM fungus inoculation and low P (available for plants) levels in substrates. The highly effective *Rhizophagus irregularis* RCAM00320 strain (formerly known as CIAM8 *Glomus intraradices* Schenck & Smith, from the collection at ARRIAM (All-Russia Research Institute for Agricultural Microbiology) was used to inoculate *M. lupulina* plants. The authors recently carried out refined identification of this strain [71]. Since AM fungi are obligate symbionts, the strain as well as the entire collection of AM fungi were grown in an accumulative culture of *Plectranthus australis* R.Br. (synonyms: Swedish Ivy, *P. verticillatus* Druce, *P. nummularius* Briq.) under conditions of low P*_i_* level in substrate at the Laboratory of Ecology of Symbiotic and Associative Rhizobacteria, ARRIAM [72].

### 5.2. Microvegetation Method

A.P. Yurkov et al. earlier described the plant cultivation protocol details using phytobox [70,73]. Plants were planted with two seedlings in one vessel filled with a soil–sand mixture weighing 210 g. Agrochemical soil characteristics: sod-podzolic loam-poor soil with a low P content; P_2_O_5_ and K_2_O contents (calculated using the Kirsanov reaction) were 23 mg and 78 mg per 1 kg of soil, respectively; organic matter content was 3.64%; pH_KCl_ was 6.44 and pH_H2O_ was 7.28. The first recording was performed on the 21st day after sowing during the development stage of the second true leaf. Subsequent recordings were carried out at the key stages of plant development. The final stage, flowering initiation, was estimated on the 57th day after sowing (Table 1).

### 5.3. Evaluation of Mycorrhization Parameters

During microscopic analysis of AM development, the authors used the method of macerating and staining the root samples, which was developed by J.M. Phillips and D.S. Hayman to evaluate AM infections in leguminous plant roots [74]. The method also included staining the roots with a trypan blue solution. For microscopic analysis of AM structures in crushed preparation, the authors used dried, macerated and cut (1 cm long) Medicago roots for analysis by light microscopy (Mikmed-2 var.2 microscope, LOMO, Russia; characteristics: eyepiece × 10, lens × 10, front lens in the flange of the binocular adjustment × 1.0). The estimated length of the microscope field of view was 1.69 mm. Subsequently, mycorrhization indices were calculated [75]: *F* (the frequency of mycorrhizal infection in the roots), *a* and *b* (the abundance of arbuscules and vesicles in the mycorrhized parts of roots, respectively). The minimum biological replication in each of studied variants was eight.

### 5.4. Evaluation of AM Symbiotic Efficiency

AM efficiency (mycorrhizal growth response) was calculated as the fresh weight of the aerial parts and roots of the plants, using Odum’s formula:

E = ([+АМ;] − [without AМ;]) × 100%/[without АМ;](1)

where E is the AM symbiotic efficiency; [+АМ] is the value of mycorrhized plant fresh weight; [without АМ] is the value of the fresh weight of plants without AM.

### 5.5. RNA Isolation and Expression Evaluation for Genes of Interest

Total RNA from plant material was isolated using the Trizol method [76]. PCR was performed with ubiquitin primers (Ubi-f: ATG CGA TYT TTG TGA AGA C and Ubi-r: ACC ACC ACG RAG ACG GAG) and isolated RNA, then PCR was performed after treatment with DNase, and negative results were obtained: DNA absent in RNA probes. The cDNA synthesis was performed using the Maxima First Strand cDNA Synthesis Kit with dsDNase according to the producer’s protocol (Thermo Scientific, Waltham, MA, USA). Approximately 1 μg of the total RNA was selected for synthesis. Genes of interest were selected based on an analysis of the literature [33,77]. Ten homologues of the phosphorus transport and carbohydrate metabolism genes in *M. truncatula* were selected. The “Ml” prefix was used in gene names to take *M. lupulina* species into consideration. Thus, *MlPT1*, *MlPT2*, *MlPT4*, *MlATP1* phosphorus transport genes, and *MlHXK1*, *MlSUS*, *MlSUS2*, *MlSUC4*, *MlRUB*, and *MlSTP13* carbohydrate metabolism genes were selected for analysis. Between two and three pairs of primers (synthesized in Evrogen, Moscow, Russian Federation) were chosen for all genes of interest. From these primers, the authors selected the effective pairs, which were tested in PCR. The effective pairs of selected primers for the genes of interest are presented in Table 2.

Changes in gene expression were studied by the qPCR-RT method with a BioRad CFX-96 thermal cycler (BioRad, Hercules, CA, USA) and using the reagent kit to carry out qPCR-RT in the presence of SYBR Green I. The amplification cycle parameters are: +95 °C, 10 min, 1 cycle; +95 °C, 15 s, +60 °C, 30 s, +72 °C, 30 s, 40 cycles. Amplification specificity was assessed by melting curve analysis. Changes in the expression level for the gene of interest in the experiment with AM were comparable to the expression level of this gene in the control without AM, the analysis for which was performed using the comparative 2^−∆∆CT^ method (at the beginning, the normalization of the reference gene, beta actin, was carried out, followed by the normalization for the control sample without AM). Beta actin (*β-actin*), glyceraldehyde-3-phosphate dehydrogenase (*GAPDH*), translation elongation factor 1-alpha (*EF1*) are known as commonly used reference genes [38,78,79]. According to the results of a comparative analysis, *β-actin* is the most appropriate reference gene for expression level stability, depending on the concentration of cDNA. *GAPDH* and *EF1* showed less stable expression (compared to *β-actin*) in roots and leaves when the cDNA concentration was changed. The PCR mixture (10 μL) contained 1 μL of 10× buffer B + SYBR Green, 1 μL of 2.5 mM dNTP, 1 μL of MgCl_2_ (25 mM), 0.3 μL of each pair of primers (10 mM per primer), 0.125 μL (0.625 U) of SynTaq DNA polymerase, 4.275 μL of ddH_2_O, and a 2 μL cDNA sample. The relative values of gene expression for each sample were evaluated. Biological replication was three, and technical replication was taken in three measurements (3 qPCR-RT reactions).

### 5.6. Statistical Analysis

One-way ANOVA and the Tukey’s HSD test (*p* < 0.05) as a post-hoc test were used to compare differences in AM efficiency and mycorrhization parameters at different stage of plant development. The Student’s *t*-test (*p* < 0.05 is the presence of significant differences) was used to estimate the significance of differences in the mean values of gene expression levels between the variants with AM and without AM (in control).

## Figures and Tables

**Table 1:** The plant development stages in *Medicago lupulina* under conditions of low phosphorus level in substrate and with/without AM inoculation.

Analysis No	Day after Sowing and Inoculation (DAS)	Host Plant Development Stage	
		without AM	+AM
1	21	2nd leaf initiation (2LI)	2nd leaf development (2L)
2	27	2nd leaf development (2L)	shooting initiation, 3rd leaf (3L)
3	33	shooting initiation, 3rd leaf (3L)	4th leaf, shooting (SH)
4	40	4th leaf, shooting (SH)	5th leaf development, axillary shoot branching initiation (SBI)
5	47	5th leaf development, axillary shoot branching initiation (SBI)	6–7th leaf development, axillary shoot branching (SB)
6	57	6–7th leaf development, axillary shoot branching, flowering initiation (FI)	8th leaf, flowering initiation (FI)

Note: “without AM” is the variant without AM fungus inoculation, “+AM” is the variant inoculated with *R. irregularis* AM fungus.

**Table 2:** Primer sequences for *Medicago lupulina* genes of interest which are homologues of the phosphorus transport and carbohydrate metabolism genes in *M. truncatula*.

Gene Names	Names of the Primers	Primer Sequences	Gene Bank Sequense Numbers
*MlHXK1*	HXK1-1F	CCCTGGAGAACAGATTTTTGAGA	MN737455
	HXK1-1R	CCTCAATTTGCTTCCAACCTC	
*MlPT1*	PT1-2F	TACTCACTCACATTCTTCTTTGC	MN737457
	PT1-2R	CCAAGCATGATAAGAGAGTTCTTA	
*MlPT2*	PT2-2F	CAGAACAAGGACAAGAGTAAAGC	MN737458
	PT2-2R	CCGTTTTGTTATGAGAATGAGATTG	
*MlPT4*	PT4-1F	GGGATTAGAAGTCCTTGAGGC	MN737459
	PT4-1R	GGCCACACCGGTGACTAAG	
*MlSUS*	SUS-3F	GGAGAGGCTTGATGAAACCTT	MN756680
	SUS-3R	CCAAATGCACCATCAGTCAG	
*MlSUS2*	SUS2-2F	GATACTCTTTCTGCTCACCGTA	MN737460
	SUS2-2R	GACATTAACACGGACATATTCCC	
*MlSUC4*	SUC4-2F	GGAGCTATTTGGGACATTCAG	MN737461
	SUC4-2R	CTGAATTAAGCAATAAACCAAGTGC	
*MlRUB*	RUB-1F	GGCTTCCTCTATGATCTCCTC	MN737462
	RUB-1R	GCCAATAGGAGGCCACACC	
*MlATP1*	ATP1-2F	CCTTTGTCATGGGTTATGGAAG	MN737456
	ATP1-2R	CATTTTCCGTCGCGAAGTACC	
*MlSTP13*	STP13-2F	GTTTGTGTCAACTTCCTCTTCAC	MN737463
	STP13-2R	CAATGTTGCTTCCACACTCTTTG	
*MlActin*	actin7-f2	GGCAGATGCTGAGGATATTCAA	MN756679
	actin7-r2	GTATGACGAGGTCGGCCAA	

Note: by using the presented primers, the sequenced parts of genes of interest were deposited in GeneBank. Actin7 is beta actin. All transcript lengths for applied primers ranged from 127 bp to 254 bp.
